# Short-term outcomes of non-ST segment elevation acute coronary syndrome after percutaneous coronary intervention: a single-center speckle tracking echocardiographic study in Vietnam

**DOI:** 10.3389/fcvm.2025.1619262

**Published:** 2025-10-22

**Authors:** Ha Viet Trinh, Dung Viet Nguyen, Loi Doan Do, Binh Thanh Le, Hoai Thi Thu Nguyen

**Affiliations:** ^1^ Vietnam National Heart Institute, Bach Mai Hospital, Hanoi, Vietnam; ^2^ Department of Internal Medicine, VNU-University of Medicine and Pharmacy, Hanoi, Vietnam; ^3^ Department of Cardiology, Hanoi Medical University, Hanoi, Vietnam

**Keywords:** non-ST segment elevation acute coronary syndrome, global longitudinal strain, global circumferential strain, global radial strain, prognosis, left ventricular function

## Abstract

**Background:**

Data on the prognostic value of myocardial strain in patients with non-ST-segment elevation acute coronary syndrome (NSTE-ACS) from low- and middle-income countries remain scarce. This study aimed to evaluate the prognostic significance of left ventricular myocardial strain in patients with NSTE-ACS after successful percutaneous coronary intervention (PCI) in Vietnam.

**Methods:**

In this prospective cohort study, consecutive patients diagnosed with NSTE-ACS and treated with PCI underwent conventional and speckle-tracking echocardiography within 24 h post-PCI to assess myocardial function, including global longitudinal strain (GLS), global circumferential strain (GCS), and global radial strain (GRS). Patients were followed for 12 months. The association between echocardiographic parameters and major adverse cardiovascular events (MACE) was analyzed using Kaplan–Meier survival curves and Cox proportional hazards models. Prognostic performance was assessed using receiver operating characteristic (ROC) curves, area under the curve (AUC), and other diagnostic indices.

**Results:**

A total of 127 patients were included (mean age 65.5 ± 10.5 years; 71.3% male). During 12 months of follow-up, 26 patients (20.5%) experienced MACE. The MACE group had significantly impaired GLS, GCS, and GRS compared with the event-free group (all *p* < 0.0001). In multivariable analysis, only higher (less negative) GLS remained an independent predictor of MACE (HR: 1.62; 95% CI: 1.26–2.08; *p* < 0.001). GLS demonstrated the strongest prognostic performance among echocardiographic variables, with an AUC of 0.967 (95% CI: 0.941–0.994). At the optimal cutoff, an GLS ≥ –16% demonstrated a sensitivity of 100% (95% CI: 86.8–100) and a specificity of 85.1% (95% CI: 76.7–91.4).

**Conclusion:**

In patients with NSTE-ACS, post-PCI GLS, GCS, and GRS were significantly more impaired in those who developed MACE compared with the event-free group, indicating underlying cardiac dysfunction or myocardial injury. Among these parameters, GLS emerged as an independent predictor of MACE after PCI and may serve as a valuable tool for identifying high-risk patients.

## Graphical Abstract

**Figure:**
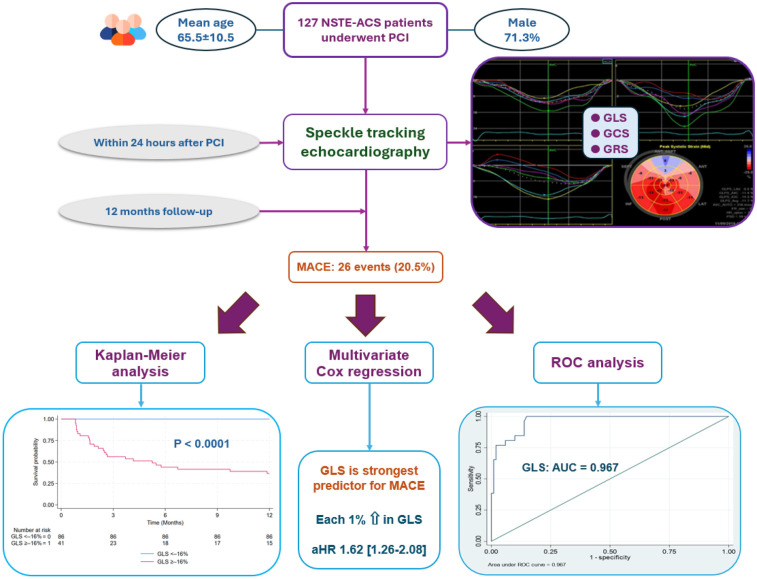

## Introduction

The proportion of non-ST-elevation myocardial infarction (NSTEMI) among total acute coronary syndrome (ACS) cases increased substantially between 2009 and 2019, with a particularly marked rise in older patients (1). This trend is attributable not only to the widespread adoption of high-sensitivity troponin assays, which enable earlier and more accurate detection of myocardial injury, but also to the growing burden of cardiovascular comorbidities in the aging population (1). In Vietnam, among the total number of patients undergoing coronary artery intervention at a single center, the intervention rate in non–ST-segment elevation acute coronary syndromes (NSTE-ACS) accounted for 39.7%, in which the prevalence of NSTEMI and unstable angina (UA) was 16.2% and 23.7% (2), similar to findings from Thailand's national percutaneous coronary intervention (PCI) registry study (3).

Despite substantial advances in diagnosis and treatment, mortality among patients with NSTE-ACS remains unacceptably high (4). Numerous functional and morphological parameters of the left ventricle are correlated with unfavorable outcome in ACS (5, 6). In particular, left ventricular (LV) function is an important prognostic predictor for ACS patients (6). Current guidelines recommend that left ventricular ejection fraction (LVEF) be determined before hospital discharge in patients with ST-elevation acute coronary syndrome (STE-ACS) and NSTE-ACS (4, 7, 8).

Speckle tracking echocardiography offers quantitative measurement of LV systolic dysfunction in the early phase, even when the LVEF is within the normal range (9, 10). Personalized risk stratification remains a pivotal issue in optimizing treatment for patients with NSTE-ACS, even after successful PCI. Several studies have evaluated the prognostic value of post-PCI echocardiographic strain parameters in NSTE-ACS populations, demonstrating that these measures are independently associated with mortality, adverse left ventricular remodeling, and other major cardiac events—including myocardial infarction (MI), ventricular arrhythmias, and hospitalization for heart failure—and have good predictive ability for these outcomes (11–17). However, all of these studies were conducted in Western or high-income Asian countries, and no evidence has been reported from low- and middle-income countries (LMICs). Therefore, this study aimed to evaluate the prognostic significance of global strain parameters, including GLS, GRS, and GCS, measured by two-dimensional speckle-tracking echocardiography (2D-STE), in patients with NSTE-ACS following successful PCI in Vietnam.

## Methods

### Study design and patient population

This prospective cohort study was conducted at the Vietnam National Heart Institute, Bach Mai Hospital, Hanoi, Vietnam. Patients admitted with NSTE-ACS and successfully treated with PCI between January 2019 and January 2020 were enrolled if they met the inclusion criteria and provided signed, informed consent. The diagnosis of NSTE-ACS was established in accordance with the European Society of Cardiology (ESC) guidelines (18).

Eligibility criteria included successful PCI, defined as achieving Thrombolysis in Myocardial Infarction (TIMI) grade 3 coronary flow after the procedure. Exclusion criteria included atrial fibrillation, paced rhythm, severe aortic stenosis, and poor-quality images noted at the time of echocardiographic examination. Upon admission, hospital records were reviewed to document data on diabetes mellitus, hypertension, personal and family history of ischemic heart disease, smoking status, and pharmacological treatment. The Global Registry of Acute Coronary Events (GRACE) risk score was computed at admission using eight clinical parameters: age, heart rate, systolic blood pressure, serum creatinine level, ST-segment deviation on electrocardiogram, elevated cardiac biomarkers, Killip classification, and history of cardiac arrest (19).

**Figure 1:**
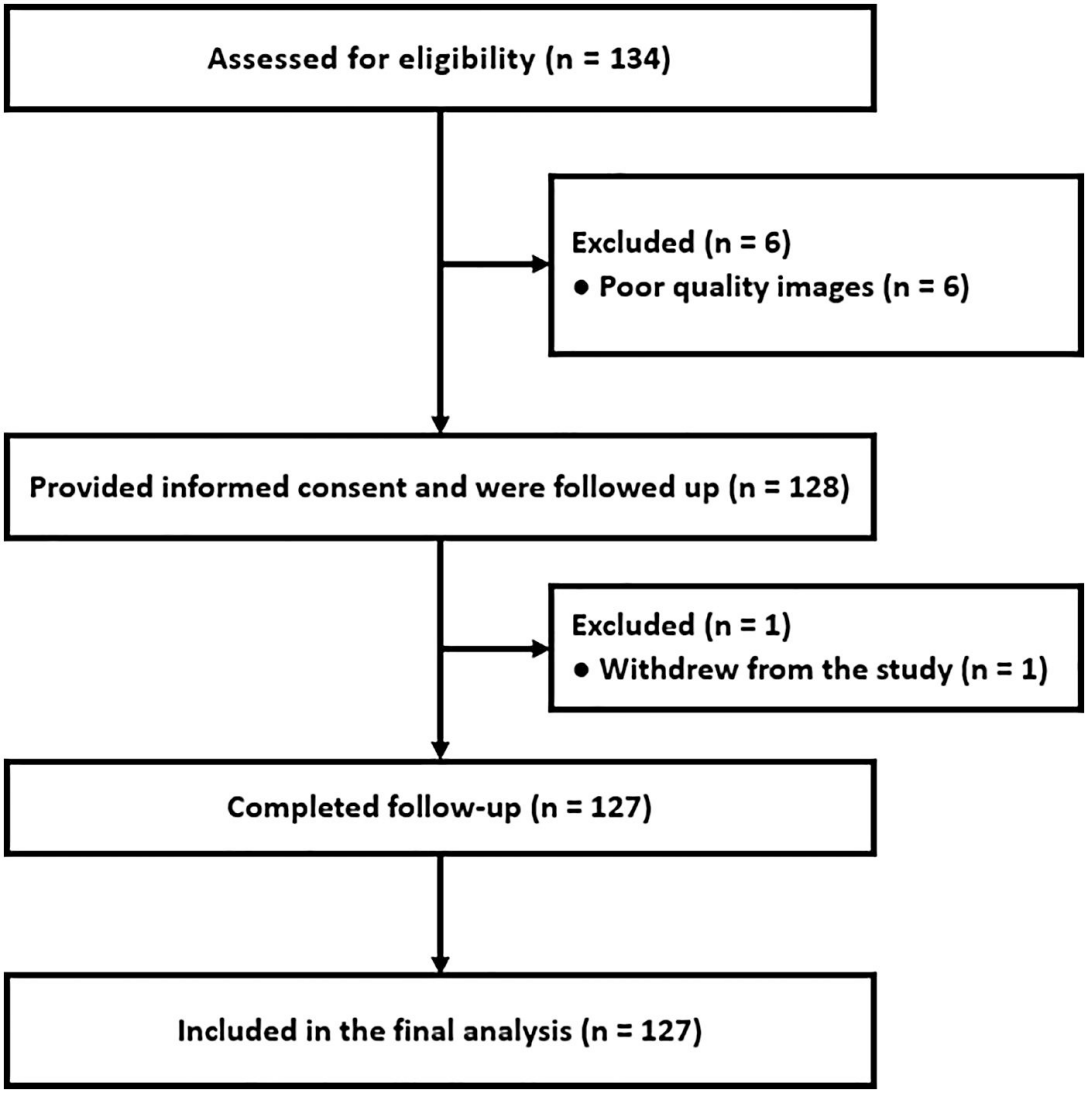
Study flowchart illustrating patient selection and follow-up.

Findings on coronary angiography were recorded (Figure 1), including the culprit lesion, the number of diseased vessels, and lesion complexity as assessed by the SYNTAX (SYNergy between PCI with TAXUS™ and Cardiac Surgery) score. Additional biochemical workup included creatinine, peak high-sensitivity troponin T (hs-Troponin T), N-terminal pro-B-type natriuretic peptide (NT-proBNP) and complete blood count during the hospital stay.

The sample size was estimated using the Hanley and McNeil method, which is commonly applied in studies assessing the diagnostic accuracy of a test based on the area under the ROC curve (AUC) (20). Calculations were performed with MedCalc Software (Version 23.3.7). From prior studies, we expected the most promising speckle-tracking echocardiographic parameter (likely GLS) to achieve an AUC of about 0.915, while setting 0.75 as the lowest acceptable AUC (21). We assumed that 18% of patients would experience adverse events within 12 months. Using a two-sided significance level of 0.05 and a statistical power of 80%, the required minimum sample size was 117 patients. To account for an anticipated 12% of patients with poor image quality or loss to follow-up, the final target sample size was increased to 133 patients.

### Echocardiography and strain analysis

Echocardiography was performed within 24 h after PCI using the Vivid E9 system (General Electric [GE] Healthcare Technologies Inc., Chicago, Illinois, USA). Imaging was conducted according to the recommendations of the American Society of Echocardiography (22–24). The frame rate during acquisition of left ventricular views was between 50 and 90 frames/s. All the views were analyzed offline with the EchoPAC software package version 113. GLS analysis involved evaluating apical two-, three-, and four-chamber views. In each of the three apical projections, three points (two at the annulus and one at the apex) were manually placed, allowing the software to semi-automatically trace the myocardium across the cardiac cycle. The region of interest was modified to encompass the myocardial thickness. Pulse wave Doppler imaging through the aortic valve was used to determine the timing of aortic valve closure.

The left ventricle was divided into 17 segments, covering the entire myocardium (25). Strain values were calculated by averaging the mean value in each segment (Figure 1). GCS and GRS were assessed from parasternal short-axis views at basal, mid, and apical levels. The short-axis region of interest was subdivided into six segments. The endocardium and epicardium were tracked manually. The anterior insertion point of the right ventricular free wall served as an anatomical landmark. The GCS and GRS were determined by averaging segmental-level measurements obtained from the same frames. All assessments were performed over three consecutive cardiac cycles and the results were averaged.

All analyses were conducted by two independent, experienced operators who were blinded to follow-up information. Final results were determined based on consensus between the two operators.

### Follow up and endpoint definition

All patients included in the study received appropriate treatment in accordance with ESC guidelines and were followed up for 12 months after PCI. The primary endpoint was major adverse cardiovascular events (MACE), defined as a composite of all-cause mortality; MI (classified according to the Fourth Universal Definition of MI) (26); stroke (defined as the sudden onset of neurological signs or symptoms consistent with a focal or multifocal vascular territory within the brain, spinal cord, or retina) (27); or hospitalization for heart failure (defined according to the uniform criteria established by the Standardized Data Collection for Cardiovascular Trials Initiative and the U.S. Food and Drug Administration) (28–30). Detailed definitions of the component outcomes are provided in the Supplemental Material.

Endpoint data were obtained through direct patient contact and rigorously verified by reviewing medical records and consulting the patients' treating physicians. Information on hospitalizations for heart failure, stroke and MI was collected through a systematic review of all hospital admission records. These events were independently adjudicated by a reviewer blinded to echocardiographic data. Causes of death were determined from hospital records and classified as cardiac or non-cardiac.

### Statistical analysis

Categorical variables were summarized as frequencies and percentages. Continuous variables were presented as mean (standard deviation [SD]) or median (interquartile range [IQR]). Differences in continuous variables between the two groups were evaluated using Student's *t*-test or the Wilcoxon rank-sum test, depending on data distribution. Differences in categorical variables were assessed using the chi-square test or Fisher's exact test, as appropriate. Unadjusted risks of MACE were estimated using Kaplan–Meier survival analysis, and intergroup differences were compared with the log-rank test. Multivariable Cox regression analysis was performed to assess the association between echocardiographic strain parameters (GLS, GCS, and GRS) and the incidence of MACE within 12 months after PCI, expressed as adjusted hazard ratios (aHRs) with 95% confidence intervals (CIs). Potential covariates included in the multivariable model were selected from prognostic factors that showed significant differences between patients with and without MACE (univariable analysis). The prognostic value of global strain measurements (GLS, GRS, and GCS) was evaluated using ROC curve analysis and AUC. The optimal cutoff value for each parameter was determined using ROC curve analysis and the Youden index, which identifies the point that maximizes the sum of sensitivity and specificity. In addition, the sensitivity, specificity, positive predictive value (PPV), negative predictive value (NPV), and overall accuracy of each parameter at their optimal cutoff values were also assessed. All statistical tests were two-tailed, and a *p*-value <0.05 was considered statistically significant. All statistical analyses and graphical plots were performed using STATA software, version 18 (StataCorp LLC, College Station, TX, USA).

### Ethics approval

Ethical approval for the study was granted by the Ethics Committee of Hanoi Medical University (Reference No. 5510/QD-DHYHN). All participants received an information sheet detailing the study objectives, procedures, and their rights. Written informed consent was obtained from each participant prior to enrollment. Patient-identifying information was anonymized and handled with strict confidentiality.

## Results

### Baseline characteristics and strain parameters

**Figure 2:**
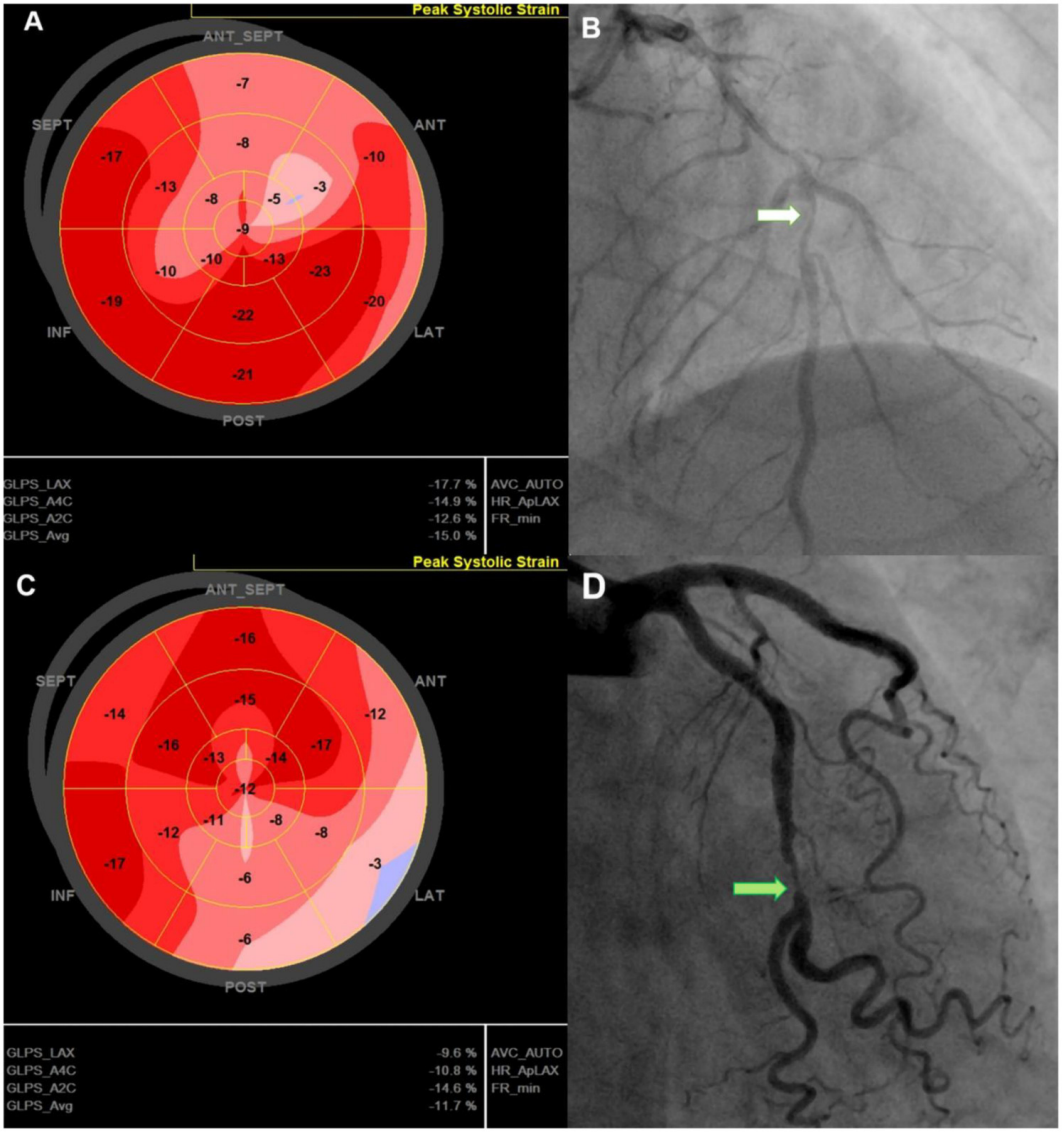
Representative cases illustrating GLS characteristics in post-PCI NSTE-ACS patients. 74-year-old man with a GLS of −15%, reduced longitudinal strain in the anterior septal region **(A)**, EF of 51%, 90% stenosis of the LAD (white arrow)—**(B)**, and no MACE during follow-up. 67-year-old man with a GLS of −11.7% with reduced strain in the lateral and posterior regions **(C)**, LVEF of 52%, and 90% stenosis of the LCx (green arrow) **(D)**; admitted for acute heart failure within 12-month follow up. GLS, Global Longitudinal Strain; LAD, left anterior descending (coronary artery); LCx, left circumflex (coronary artery); LVEF, left ventricular ejection fraction; MACE, major adverse cardiovascular events; PCI, percutaneous coronary intervention.

A total of 133 patients with NSTE-ACS were prospectively enrolled. Six were excluded due to poor image quality, and one withdrew during follow-up. No patients were lost to follow-up, resulting in 127 participants for the final analysis (Figure 2). The mean age was 65.5 ± 10.5 years, and 71.3% were men.

The baseline characteristics of patients, stratified by the occurrence of MACE (*n* = 26) vs. no MACE (*n* = 101) during the first 12 months of follow-up, are presented in Table 1. Higher GRACE scores, *E*/*e*′ ratios, left ventricular end-systolic volume (LVESV), NT-proBNP, and high-sensitivity troponin T were observed in patients who experienced adverse cardiac events, whereas LVEF was lower (*p* < 0.05). There were no significant differences in the distribution of gender, body mass index (BMI), or cardiovascular risk factors (diabetes, smoking, hypertension, dyslipidemia, and family history of coronary artery disease [CAD]) between the two groups. Baseline peak hs-troponin T levels and GRACE scores were also higher in the MACE group (*p* < 0.05). There were no differences in medical treatment at hospital discharge between patients with and without MACE: 100% received statins, aspirin, and a P2Y12 inhibitor; 89% received beta-blockers; and 94% received either an angiotensin-converting enzyme inhibitor (ACEi), angiotensin receptor blocker (ARB), or angiotensin receptor–neprilysin inhibitor (ARNI).

**Table 1:** Baseline characteristics of the study population stratified by the incidence of MACE during 12-month follow-up.

Characteristic	All patients	No MACE	MACE	*P* value
	*n* = 127	*n* = 101	*n* = 26	
Age (years), mean ± SD	65.5 ± 10.5	64.5 ± 9.9	69.5 ± 11.9	0.0299^a^
Male gender, *n* (%)	90 (70,9)	72 (71.3)	18 (69.2)	0.837^c^
BMI (kg/m^2^), mean ± SD	22.1 ± 2.6	22.3 ± 2.6	21.7 ± 2.7	0.3345^a^
Cardiovascular risk factors				
Hypertension, *n* (%)	94 (74.0)	73 (72,3)	21 (80.8)	0.379^c^
Diabetes, *n* (%)	35 (27.6)	26 (25.7)	9 (34.6)	0.367^c^
Dyslipidemia, *n* (%)	38 (29.9)	32 (31.7)	6 (23.1)	0.393^c^
Familial history of CAD, *n* (%)	20 (15.8)	16 (15.8)	4 (15.4)	1.000^d^
Smoking, *n* (%)	54 (42.5)	45 (44.6)	9 (34.6)	0.361^c^
Numbers of diseased vessels				
1 vessel, *n* (%)	46 (36.2)	43 (42.6)	3 (11.5)	0.004^c^
2 vessels, *n* (%)	42 (33.1)	33 (32.7)	9 (34.6)	
3 vessels, *n* (%)	39 (30.7)	25 (24.8)	14 (53.9)	
SYNTAX Score, median (IQR)	21.3 (10–27)	20.9 (10–23)	22.3 (18–27)	0.1603^b^
GRACE score, mean ± SD	108.8 ± 25.3	105.0 ± 22.9	123.5 ± 28.9	0.0007^a^
Creatinine (µmol/L), median (IQR)	84 (71–102)	85 (73–100)	79 (71–107)	0.9846^b^
Peak hs-Troponin T (ng/L), median (IQR)	166.8 (30–798)	140.2 (30–650)	590.5 (116.7–1,690)	0.0310^b^
NT-proBNP (pmol/L), median (IQR)	48.2 (16.0–108)	38.6 (11.2–86.6)	112.8 (48.0–262.4)	0.0008^b^
Medical Treatment at hospital admission				
Aspirin, *n* (%)	109 (85.8)	84 (83.2)	25 (96.2)	0.119^d^
P2Y12 inhibitor, *n* (%)	84 (66.1)	63 (62.4)	21 (80.8)	0.077^c^
Beta blocker, *n* (%)	88 (69.3)	68 (67.3)	20 (76.9)	0.344^c^
ACEI/ARB/ARNI, *n* (%)	48 (37.8)	41 (40.6)	7 (26.9)	0.200^c^
Statin, *n* (%)	113 (89.0)	88 (87.1)	25 (96.2)	0.298^d^
Time between NSTE-ACS diagnosis and PCI (h), median (IQR)	25 (23–29)	26 (23–30)	25 (23–27)	0.1910^b^
Medical Treatment at hospital discharge				
Aspirin, *n* (%)	127 (100)	101 (100)	26 (100)	1.000^d^
P2Y12 inhibitor, *n* (%)	127 (100)	101 (100)	26 (100)	1.000^d^
Beta blocker, *n* (%)	113 (89.0)	89 (88.1)	24 (92.3)	0.543^c^
ACEI/ARB/ARNI, *n* (%)	94 (74.0)	73 (72,3)	21 (80.8)	0.775^c^
Statin, *n* (%)	127 (100)	101 (100)	26 (100)	1.000^d^
SGLT2i, *n* (%)	32 (25.2)	25 (24.75)	7 (26.09)	0.820^c^

ACEI, Angiotensin-Converting Enzyme Inhibitor; ARB, Angiotensin II Receptor Blocker; ARNI, Angiotensin Receptor–Neprilysin Inhibitor; BMI, Body Mass Index; CAD, Coronary Artery Disease; GCS, Global Circumferential Strain; GLS, Global Longitudinal Strain; GRACE, Global Registry of Acute Coronary Events; GRS, Global Radial Strain; IQR, interquartile range; LVEDV, Left Ventricular End-Diastolic Volume; LVEF, Left Ventricular Ejection Fraction; LVESV, Left Ventricular End-Systolic Volume; NT-proBNP, N-terminal pro–B-type Natriuretic Peptide; SGLT2i, Sodium–Glucose Cotransporter-2 Inhibitors; SD, standard deviation; SYNTAX, SYNergy between PCI with TAXUS™ and Cardiac Surgery.

^a^ Student's *t*-test.

^b^ Wilcoxon rank-sum test. ^c^Chi-square test.

^d^ Fisher's exact test.

**Figure 3:**
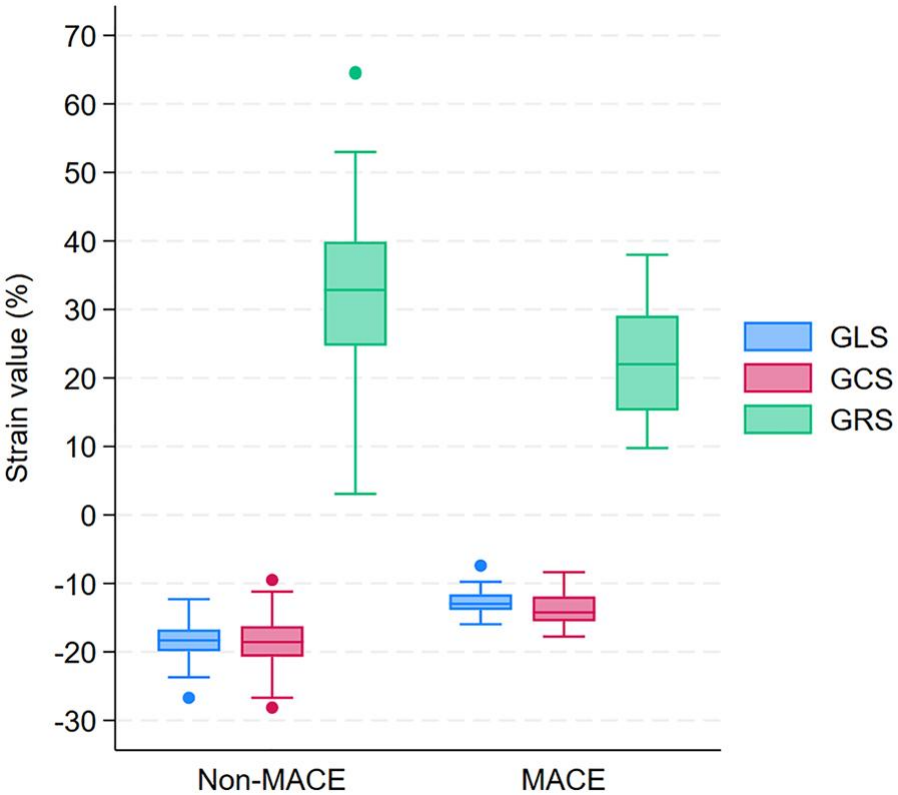
Box plots of GLS, GCS, and GRS in patients with and without MACE during 12-month follow-up after PCI. GLS, Global Longitudinal Strain; GCS, Global Circumferential Strain; GRS, Global Radial Strain; MACE, major adverse 660 cardiovascular events; PCI, percutaneous coronary intervention.

The mean post-PCI GLS for the overall cohort was −17.2 ± 3.3%. GLS and GCS were significantly higher in patients who developed MACE. Specifically, GLS was −12.8 ± 2.1% in patients with MACE compared with −18.4 ± 2.4% in those without MACE (*p* < 0.0001; Table 2 and Figure 3). Similarly, GCS was −13.6 ± 2.7% in patients with MACE and −18.5 ± 3.7% in those without MACE (*p* < 0.0001; Table 2 and Figure 3). On the other hand, post-PCI GRS was significantly lower in patients who developed MACE compared with those who did not (22.7 ± 8.1% vs. 33.1 ± 10.6%, *p* < 0.0001; Table 2 and Figure 3).

**Table 2:** Echocardiographic characteristics stratified by the incidence of MACE within 12 months of follow-up.

Measurement	All patients	No MACE	MACE	*P* value
	*n* = 127	*n* = 101	*n* = 26	
LVEF [Simpson] (%), mean ± SD	57.9 ± 8.2	60.1 ± 6.5	49.5 ± 8.6	<0.0001^a^
LVEDV (ml), median (IQR)	68 (54–79)	67 (53–78)	69 (62.5–85)	0.1903^b^
LVESV (ml), median (IQR)	27 (21–35)	25 (21–31)	37.5 (27–44)	<0.0001^b^
GLS (%), mean ± SD	–17.2 ± 3.3	–18.4 ± 2.4	–12.8 ± 2.1	<0.0001^a^
GCS (%), mean ± SD	–17.4 ± 4.0	–18.5 ± 3.7	–13.6 ± 2.7	<0.0001^a^
GRS (%), mean ± SD	30.8 ± 11.0	33.1 ± 10.6	22.7 ± 8.1	<0.0001^a^
E/e’, median (IQR)	10 (8.1–11.8)	9.4 (7.9–11.3)	11.9 (10.5–15.7)	<0.0001^b^
E/A, median (IQR)	0.73 (0.65–0.85)	0.73 (0.65–0.84)	0.71 (0.66–0.95)	0.8387^b^

GCS, Global Circumferential Strain; GLS, Global Longitudinal Strain; GRACE, Global Registry of Acute Coronary Events; GRS, Global Radial Strain; IQR, interquartile range; LVEDV, Left Ventricular End-Diastolic Volume; LVEF, Left Ventricular Ejection Fraction; LVESV, Left Ventricular End-Systolic Volume; MACE, Major Adverse Cardiovascular Events; SD, standard deviation.

^a^ Student's *t*-test.

^b^ Wilcoxon rank-sum test.

### Predictor of cardiovascular events

During the 12-month follow-up, 26 patients (20.5%) experienced major adverse cardiovascular events, comprising 3 deaths, 9 nonfatal MIs, 12 hospitalizations for heart failure, and 2 strokes. In multivariable analysis, GCS and GRS were no longer significant (aHR: 1.15 [0.98–1.34], *p* = 0.083; and 0.99 [0.93–1.06], *p* = 0.798), whereas GLS remained independently associated with MACE. Specifically, each 1% increase in the GLS value was associated with a 1.62-fold increased risk of MACE within 12 months after PCI (aHR: 1.62; 95% CI: 1.26–2.08; *p* < 0.001; Table 3).

**Table 3:** Multivariable Cox regression analysis assessed the association between echocardiographic strain parameters and the incidence of MACE within 12 months after PCI.

Variable	Multivariable analysis	*p*-value
	aHR (95% CI)	
Age (increase 1 year)	0.97 (0.89–1.05)	0.447
Multivessel disease (Yes)	2.60 (0.64–10.46)	0.179
Log (Peak hs-Troponin T) (increase 1)	1.12 (0.82–1.54)	0.481
Log (NT-proBNP) (increase 1)	1.03 (0.68–1.54)	0.903
GRACE score (increase 10 point)	1.003 (0.71–1.41)	0.985
LVESV (ml)	0.99 (0.95–1.03)	0.687
E/e’ (increase 1)	1.07 (0.90–1.27)	0.415
LVEF (decrease 1%)	0.98 (0.91–1.06)	0.640
GLS (increase 1%)	1.62 (1.26–2.08)	<0.001
GCS (increase 1%)	1.15 (0.98–1.34)	0.083
GRS (decrease 1%)	0.99 (0.93–1.06)	0.798

aHR, adjusted hazard ratio; CI, confidence interval; GCS, Global Circumferential Strain; GLS, Global Longitudinal Strain; GCS, Global Circumferential Strain; GRACE, Global Registry of Acute Coronary Events; GRS, Global Radial Strain; LVEDV, Left Ventricular End-Diastolic Volume; LVEF, Left Ventricular Ejection Fraction; LVESV, Left Ventricular End-Systolic Volume; MACE, Major Adverse Cardiovascular Events; NT-proBNP, N-terminal pro–B-type Natriuretic Peptide.

**Figure 4:**
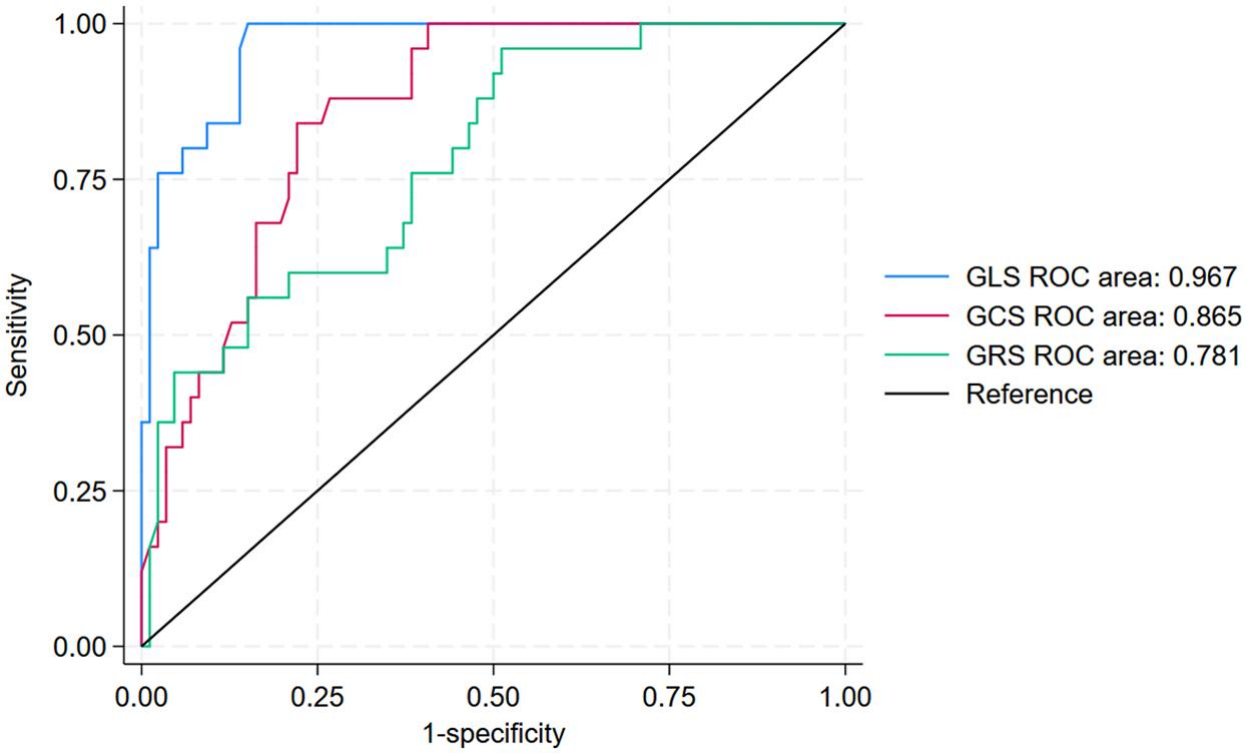
ROC curves of GLS, GCS, and GRS for 12-month MACE prediction after PCI. GLS, Global Longitudinal Strain; GCS, Global Circumferential Strain; GRS, Global Radial Strain; MACE, major adverse cardiovascular events; PCI, percutaneous coronary intervention.

**Figure 5:**
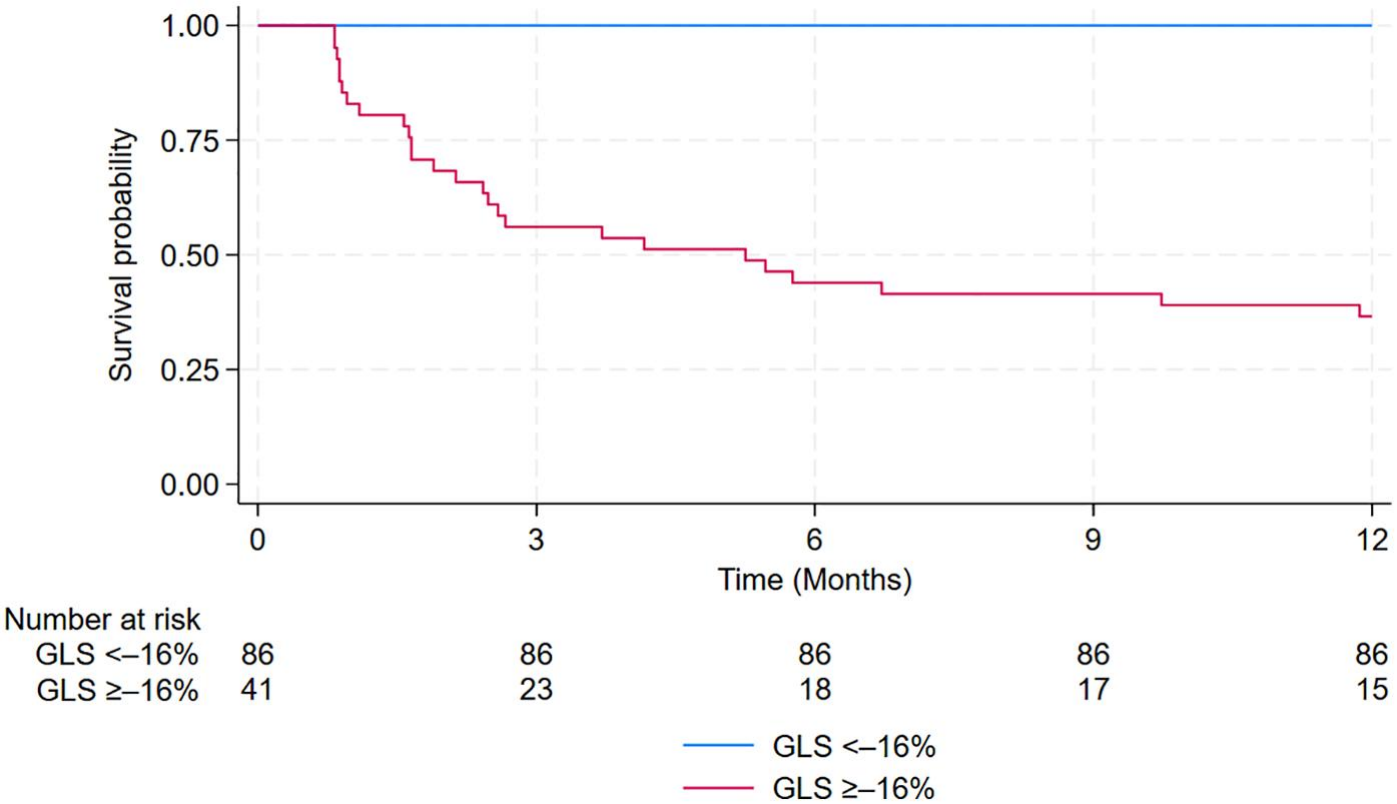
Kaplan–Meier curves estimating the cumulative incidence of MACE after PCI in patients with GLS ≥0%–16% vs. those with GLS **<**–16%. GLS, Global Longitudinal Strain; MACE, major adverse cardiovascular events; PCI, percutaneous coronary intervention.

The prognostic value of GLS, GCS, and GRS in predicting the incidence of MACE within 12 months after PCI among patients with NSTE-ACS is shown in Figure 4 and Table 4. In ROC analysis, GLS demonstrated strong discriminative ability, with an AUC of 0.967 (95% CI: 0.941–0.994), whereas GCS and GRS showed lower AUCs of 0.865 (95% CI: 0.798–0.931) and 0.781 (95% CI: 0.683–0.880), respectively. Using the optimal cut-off values (GLS ≥–16%, GCS ≥–16.1%, and GRS ≤33.5%), GLS consistently demonstrated superior prognostic performance compared with GCS and GRS. Specifically, GLS showed a sensitivity of 100% (95% CI: 86.8–100), specificity of 85.1% (95% CI: 76.7–91.4), positive predictive value (63.4%, 95% CI: 46.9–77.9), negative predictive value (100%, 95% CI: 95.8–100), and overall accuracy of 88.2% (95% CI: 81.4–92.7). In addition, Kaplan–Meier survival analysis showed that patients with a post-PCI GLS ≥–16% had a significantly higher cumulative incidence of MACE compared with those with a post-PCI GLS <–16% (*p* < 0.0001; Figure 5).

**Table 4:** Diagnostic value of GLS, GCS, and GRS in predicting 12-month MACE after PCI.

Prediction performance metric	GLS	GCS	GRS
AUC (95% CI)	0.967 (0.941–0.994)	0.865 (0.798–0.931)	0.781 (0.683–0.880)
Cut-off	–16.0%	–16.1%	33.5%
Sensitivity (% [95% CI])	100 *(*86.8–100)	80.8 *(*60.6–93.4)	96.0 *(*79.6–99.9)
Specificity (% [95% CI])	85.1 *(*76.7–91.4)	77.5 *(*67.4–85.7)	48.8 *(*37.9–59.9)
PPV (% [95% CI])	63.4 *(*46.9–77.9)	51.2 *(*35.1–67.1)	35.3 *(*24.1–47.8)
NPV (% [95% CI])	100 *(*95.8–100)	93.2 *(*84.9–97.8)	97.7 *(*87.7–99.9)
Accuracy (% [95% CI])	88.2 *(*81.4–92.7)	78.3 *(*70.7–85.8)	59.5 *(*50.2–68.1)

GLS, Global Longitudinal Strain; GCS, Global Circumferential Strain; GRS, Global Radial Strain; MACE, major adverse cardiovascular events; NPV, negative predictive value; PPV, positive predictive value; PCI, percutaneous coronary intervention.

## Discussion

To the best of our knowledge, this is the first study to evaluate the prognostic value of left ventricular myocardial strain in patients with NSTE-ACS following successful PCI in Vietnam. Our findings demonstrated that post-PCI GLS was independently associated with the incidence of MACE during the 12-month follow-up (aHR per 1% increase in GLS: 1.62, 95% CI: 1.26–2.08) and provided the strongest predictive value among strain parameters. Specifically, GLS yielded an AUC of 0.967 (95% CI: 0.941–0.994) with a sensitivity of 100% (95% CI: 86.8–100), specificity of 85.1% (95% CI: 76.7–91.4), positive predictive value of 63.4% (95% CI: 46.9–77.9), negative predictive value of 100% (95% CI: 95.8–100), and overall accuracy of 88.2% (95% CI: 81.4–92.7) at the GLS cut-off value of −16%. Compared with post-PCI GCS and GRS, which demonstrated lower discriminative ability, post-PCI GLS consistently outperformed both parameters, reinforcing its role as the most robust predictor of adverse cardiovascular outcomes in this population.

Although GLS, GCS, and GRS are all derived from 2D-STE to characterize myocardial shortening or lengthening relative to the cardiac cycle and quantify myocardial deformation as a percentage—thus providing a more sensitive assessment of systolic function than conventional measures such as LVEF—our study demonstrated that only GLS was independently associated with the risk of MACE within 12 months after PCI in patients with NSTE-ACS (31). Although no studies to date have specifically evaluated the prognostic significance of GCS and GRS in relation to MACE among patients with NSTE-ACS, investigations conducted in STEMI populations by Iwahashi et al. (32) and Chu et al. (33), which assessed GLS, GCS, and GRS simultaneously, reported findings consistent with ours. The superior prognostic performance of GLS may be explained by the fact that subendocardial fibers, which are primarily responsible for longitudinal shortening, are more susceptible to ischemia and therefore exhibit earlier alterations after NSTE-ACS compared with mid-wall circumferential or transmural radial fibers (31). By reflecting subendocardial longitudinal dysfunction, GLS is able to detect subtle impairments in left ventricular function earlier and more accurately than other echocardiographic strain parameters (31).

The prognostic significance of echocardiographic global strain parameters in patients with ACS has been evaluated in several previous studies, with results broadly consistent with our findings, particularly regarding the independent association of GLS with adverse cardiovascular outcomes. Ersbøll et al. (34) evaluated the prognostic value of GLS (echocardiography was performed within 48 h of admission) in 849 patients with acute MI and preserved LVEF, demonstrating that GLS was an independent predictor of the composite endpoint of all-cause mortality and hospitalization for heart failure, with an adjusted hazard ratio of 1.14 (95% CI: 1.04–1.26). Similarly, in a prospective cohort study of 310 patients with NSTE-ACS and a follow-up duration of 24 months conducted in Romania, Ionac et al. demonstrated that GLS (echocardiography was performed at the time of hospital discharge) remained an independent predictor of adverse cardiovascular events (cardiac mortality, malignant ventricular arrhythmias, hospital readmission for heart failure, and reinfarction) after adjustment for covariables (aHR per 1% increase in GLS: 1.01, 95% CI: 0.950–1.074) (14). A large study from Germany including 729 patients with NSTEMI reported by Backmann et al. in 2024 showed that GLS, when measured within 1 year after revascularization, was independently associated with mortality (aHR per 1 SD increase in GLS: 1.47, 95% CI: 1.17–1.85) over a median follow-up of 1.5 years (IQR: 0.5–4.2 years) (16). More recently, Lenell et al., in a multicenter study of 941 ACS patients with a median follow-up of 6.2 years, confirmed that GLS measured within 72 h of admission remained an independent predictor of the composite outcome after adjustment for baseline characteristics and LVEF (aHR per 1% increase in GLS: 1.068, 95% CI: 1.017–1.121) (11).

Similar to the strong performance of GLS in predicting the incidence of MACE within 12 months after PCI observed in our study, Kumar et al., in a cohort of 157 patients with acute MI, also reported excellent discriminatory ability of baseline GLS, with an AUC of 0.915 for predicting 6-month mortality after hospital admission (21). In contrast, the studies by Ionac et al. (in NSTE-ACS patients after PCI) and Lenell et al. (in ACS patients) demonstrated only modest prognostic value of GLS for adverse cardiac events, with AUCs of 0.652 (95% CI: 0.587–0.717) and 0.656 (95% CI: 0.609–0.703), respectively (11, 14). The variability in these findings may be attributable to differences in study population characteristics, variations in the definition of outcomes of interest, and the timing of echocardiographic assessment of GLS. Our results suggest that performing speckle-tracking echocardiography within 24 h after PCI may represent the most appropriate time window for prognostic evaluation in patients with NSTE-ACS.

To the best of our knowledge, apart from AUC values, no prior studies have reported detailed diagnostic indices (e.g., sensitivity, specificity, PPV, NPV, and overall accuracy) for the prognostic performance of echocardiographic GLS, GCS, and GRS in predicting MACE among patients with NSTE-ACS after PCI. Nevertheless, findings from several studies conducted in STEMI populations have been consistent with our results, consistently highlighting the strong prognostic value of GLS. For example, Lacalzada et al. reported in a cohort of 103 STEMI patients treated with primary PCI that GLS, measured within the first 48 h after PCI, with a cutoff value of −9.27%, predicted the development of combined adverse events (death, recurrent MI, heart failure, and coronary revascularization) with a sensitivity of 62.5% (95% CI: 30.6–86.3), specificity of 98.6% (95% CI: 92.3–99.8), positive predictive value of 83.3%, and negative predictive value of 95.8% (35). Similarly, in a prospective study of 216 STEMI patients undergoing primary PCI, Chu et al. demonstrated that GLS measured within 48 h, with a cutoff of −12.3%, predicted adverse events with a sensitivity of 95.7% and a specificity of 67.0% (33). More recently, Ravenna et al. evaluated 117 STEMI patients and showed that a GLS cutoff of ≤12% (measured prior to discharge) predicted all-cause mortality within 6 months after PCI with 89% sensitivity and 70% specificity (36).

Compared to conventional echocardiography, which relies on subjective visual assessment of wall motion, STE offers a quantitative and more sensitive evaluation of myocardial function (9). Myocardial deformation impairment without reduced LVEF is a common scenario that should be identified as subclinical left ventricular dysfunction (9). By analyzing GLS, STE can detect subtle, new regional dysfunction that indicates periprocedural MI, often before changes are visually apparent (9). This is crucial, as recent evidence has shown that such events are associated with significantly worse long-term prognosis in these patients (37). Moreover, several studies have shown that speckle-tracking echocardiography accurately predicts global functional recovery and left ventricular remodeling after myocardial infarction, with performance comparable to cardiac magnetic resonance (35, 38, 39). Combined with prior evidence supporting its role in myocardial deformation analysis for viability assessment in chronic ischemic left ventricular dysfunction, these findings underscore the potential of speckle-tracking to help identify patients who may or may not benefit from revascularization in borderline clinical scenarios, such as late presentation or chronic total occlusion (38). GLS, derived from 2D-STE, is software-driven and less dependent on operator expertise. Its ease of measurement and consistent results make it particularly suitable for LMICs, where challenges in training and standardization persist. The assessment of GLS using 2D-STE is now clinically feasible and should be incorporated into standard myocardial function evaluations for patients following NSTE-ACS to enhance risk stratification, particularly when LVEF is within the intermediate or normal range (40).

## Limitations

This study has several limitations. First, it was conducted at a single tertiary care center in Vietnam, which may limit the representativeness of the study population. The clinical profile and management practices at a high-volume national referral hospital may not fully reflect those of other institutions, particularly community hospitals. Second, the relatively small sample may reduce the statistical power of analyses and increase the risk of overestimating the strength of associations. Third, although multivariable adjustments were performed, the possibility of residual confounding cannot be excluded. Factors such as medication adherence, comorbidities, or unmeasured lifestyle variables may have influenced outcomes but were not fully accounted for. Finally, the findings may not be directly generalizable to populations outside Vietnam or the broader Asian region. Differences in ethnicity, cardiovascular risk profiles, health system resources, and treatment strategies may limit the applicability of these results to other settings. Larger, multicenter, and more diverse studies are warranted to confirm the prognostic value of myocardial strain in patients with NSTE-ACS across different healthcare environments.

## Conclusions

Post-PCI GLS is an independent predictor of MACE in patients with NSTE-ACS during the first year after successful PCI. By providing a more sensitive assessment of myocardial function than conventional echocardiographic indices, post-PCI GLS enhances clinicians' ability to identify high-risk patients who may benefit from closer surveillance and individualized management strategies. Its non-invasive nature, reproducibility, and strong prognostic performance further support GLS as a valuable tool for cardiovascular risk stratification, particularly in resource-limited settings such as Vietnam.

## Data Availability

The original contributions presented in the study are included in the article/Supplementary Material, further inquiries can be directed to the corresponding author.
