# Supplementary material for: Short-term outcomes of non-ST segment elevation acute coronary syndrome after percutaneous coronary intervention: a single-center speckle tracking echocardiographic study in Vietnam
Source: Front Cardiovasc Med. 2025 Oct 22;12:1619262. doi: 10.3389/fcvm.2025.1619262 (PMC12587297; doi:10.3389/fcvm.2025.1619262)
Supplement: Supplementary file 1 [file Datasheet1.pdf]

Supplementary Materials

I. Definition of Component Outcomes

1. Causes of Death

- All-Cause death was adjudicated, as either cardiac or non-cardiac death.

- Death from cardiovascular causes was defined as sudden cardiac death; death due to acute myocardial infarction, heart failure, or cardiogenic shock; other cardiovascular causes; or any death of unknown cause in the absence of a clearly established non-cardiac etiology .

2. Myocardial Infarction

- The definition of myocardial infarction used in this trial is adapted from the Fourth Universal Definition of Myocardial Infarction.

- Clinical criteria for Myocardial Infarction: The clinical definition of MI denotes the presence of acute myocardial injury detected by abnormal cardiac biomarkers in the setting of evidence of acute myocardial ischemia.

- Criteria for Myocardial Injury: Detection of an elevated cardiac troponin (cTn) value above the 99th percentile upper reference limit (URL) is defined as myocardial injury. The injury is considered acute if there is a rise and/or fall of cTn values.

2.1. Type 1 myocardial infarction (spontaneous myocardial infarction)

Detection of a rise and/or fall of cTn values with at least 1 value above the 99th percentile URL and with at least 1 of the following:

• Symptoms of acute myocardial ischemia.

• New ischemic electrocardiogram (ECG) changes.

• Development of pathological Q waves.

• Imaging evidence of new loss of viable myocardium or new regional wall motion abnormality in a pattern consistent with an ischemic etiology.

• Identification of a coronary thrombus by angiography including intracoronary imaging or by autopsy.

2.2. Type 2 myocardial infarction (myocardial infarction secondary to an ischemic imbalance)

Detection of a rise and/or fall of cTn values with at least 1 value above the 99th percentile URL, and evidence of an imbalance between myocardial oxygen supply and demand unrelated to acute coronary atherothrombosis, requiring at least 1 of the following:

• Symptoms of acute myocardial ischemia.

• New ischemic ECG changes.

• Development of pathological Q waves.

• Imaging evidence of new loss of viable myocardium or new regional wall motion abnormality in a pattern consistent with an ischemic etiology.

2.3. Type 3 myocardial infarction (myocardial infarction resulting in death)

Patients who suffer cardiac death, with symptoms suggestive of myocardial ischemia accompanied by presumed new ischemic ECG changes or ventricular fibrillation but die before blood samples for biomarkers can be obtained, or before increases in cardiac biomarkers can be identified, or MI is detected by autopsy examination.

2.4. Type 4a myocardial infarction (percutaneous coronary intervention-related myocardial infarction ≤48 hours after the index procedure)

Coronary intervention–related myocardial infarction is arbitrarily defined by an elevation of cTn values >5 times the 99th percentile URL in patients with normal baseline values. In patients with elevated pre-procedure cTn in whom the cTn level are stable (≤20% variation) or falling, the post-procedure cTn must rise by >20%. However, the absolute postprocedural value must still be at least 5 times the 99th percentile URL. In addition, 1 of the following elements is required:

• New ischemic ECG changes.

• Development of new pathological Q waves*.

• Imaging evidence of new loss of viable myocardium or new regional wall motion abnormality in a pattern consistent with an ischemic etiology.

• Angiographic findings consistent with a procedural flow-limiting complication such as coronary dissection, occlusion of a major epicardial artery or a side branch occlusion/thrombus, disruption of collateral flow, or distal embolization.

2.5. Type 4b myocardial infarction (myocardial infarction related to stent/scaffold thrombosis)

A subcategory of percutaneous coronary intervention-related myocardial infarction is stent/scaffold thrombosis, type 4b myocardial infarction, as documented by angiography or autopsy using the same criteria utilized for type 1 myocardial infarction.

2.6. Type 4c myocardial infarction (restenosis associated with percutaneous coronary intervention)

This percutaneous coronary intervention-related myocardial infarction type is designated as type 4c myocardial infarction, defined as focal or diffuse restenosis, or a complex lesion associated with a rise and/or fall of cTn values above the 99th percentile URL applying, the same criteria utilized for type 1 myocardial infarction.

2.7. Type 5 myocardial infarction (coronary artery bypass grafting surgery-related myocardial infarction ≤48 hours after the index procedure)

Coronary artery bypass grafting surgery-related myocardial infarction is arbitrarily defined as elevation of cTn values >10 times the 99th percentile URL in patients with normal baseline cTn values. In patients with elevated pre-procedure cTn in whom cTn levels are stable (≤20% variation) or falling, the post-procedure cTn must rise by >20%. However, the absolute postprocedural value still must be >10 times the 99th percentile URL. In addition, 1 of the following elements is required:

• Development of new pathological Q waves.

• Angiographic documented new graft occlusion or new native coronary artery occlusion.

• Imaging evidence of new loss of viable myocardium or new regional wall motion abnormality in a pattern consistent with an ischemic etiology.

3. Hospitalization for heart failure

A hospitalization for heart failure was defined according to the uniform criteria established by the Standardized Data Collection for Cardiovascular Trials Initiative and the U.S. Food and Drug Administration (FDA), and required the following components:

1) The adjudicated primary diagnosis is admission to hospital for heart failure.

2) The patient's length-of-stay in hospital extends for at least 12 hours (or a change in calendar date if the hospital admission and discharge times are unavailable). Emergency room visit for ≥ 12 hours with intravenous therapy would be considered equivalent as admission to hospital.

3) The patient exhibits documented new or worsening symptoms due to HF on presentation, including at least ONE of the following:

a. Dyspnea (dyspnea with exertion, dyspnea at rest, orthopnea, paroxysmal dyspnea)

b. Decreased exercise tolerance

c. Fatigue

d. Other symptoms of worsened end-organ perfusion such as dizziness, mental confusion or volume overload such as weight gain or lower extremity swelling.

4) The patient has objective evidence of new or worsening HF, consisting of at least TWO physical examination findings OR one physical examination finding and at least ONE laboratory criterion), including:

a. Physical examination findings considered to be due to heart failure, including new or worsened:

i. Peripheral edema

ii. Increasing abdominal distention or ascites (in the absence of primary hepatic disease)

iii. Pulmonary rales/crackles/crepitations

iv. Increased jugular venous pressure and/or hepatojugular reflux

v. S3 gallop

vi. Clinically significant or rapid weight gain thought to be related to fluid retention

b. Laboratory evidence of new or worsening HF, if obtained within 24 hours of presentation, including:

c. Increased B-type natriuretic peptide (BNP)/ N-terminal pro-BNP (NT proBNP) concentrations consistent with decompensation of heart failure. In patients with chronically elevated natriuretic peptides, a significant increase should be noted above baseline.

d. Radiological evidence of pulmonary congestion.

e. Non-invasive diagnostic evidence of clinically significant elevated left- or right-sided ventricular filling pressure or low cardiac output. For example, echocardiographic criteria could include: E/e' >15 or D-dominant pulmonary venous inflow pattern, plethoric inferior vena cava with minimal collapse on inspiration, or decreased left ventricular outflow tract (LVOT) minute stroke distance (time velocity integral (TVI) **OR**

f. Invasive diagnostic evidence with right heart catheterization showing a pulmonary capillary wedge pressure (pulmonary artery occlusion pressure) ≥18 mmHg, central venous pressure ≥12 mmHg, or a cardiac index <2.2 L/min/m^2.

5) The patient receives initiation or intensification of treatment specifically for HF, including at least ONE of the following:

a. Augmentation in oral diuretic therapy. NOTE: If the intensification is solely oral diuretics, the duration of hospitalization must be at least 24 hours.

b. Intravenous diuretic or vasoactive agent (e.g., inotrope, vasopressor, or vasodilator)

c. Mechanical or surgical intervention, including:

i. Mechanical circulatory support (e.g., intra-aortic balloon pump, ventricular assist device, extracorporeal membrane oxygenation, total artificial heart)

ii. Mechanical fluid removal (e.g., ultrafiltration, hemofiltration, dialysis).

An endpoint heart failure hospitalization requires admission to the hospital for heart failure, worsening of symptoms of heart failure, a duration of hospitalization of at least 12 hours, the intensification of heart failure therapy, and a committee consensus that the hospitalization was primarily due to worsening heart failure. Changes in physical signs or laboratory tests, whenever available and documented, will be considered to be supportive.

4. Stroke

Stroke is defined as an acute symptomatic episode of neurological dysfunction, more than 24 hours in duration in the absence of therapeutic intervention or death, due to cerebral, spinal or retinal tissue injury as evidenced by neuroimaging or lumbar puncture. It includes the following subclassifications:

• Ischemic stroke: infarction due to prolonged ischemia. Causes include (but are not limited to) arterial and venous thrombosis, embolism, and systemic hypoperfusion.

• Hemorrhagic stroke: caused by a non-traumatic intraparenchymal, intraventricular or subarachnoid hemorrhage.

• Undetermined: stroke with insufficient information to determine ischemic or hemorrhagic cause.

• Transient ischemic attack is a transient episode of neurological dysfunction (<24 hours) caused by temporary cerebral, spinal or retinal ischemia with no evidence of acute infarction on neuroimaging.
